# Immunoglobulin G1 subclass responses can be used to detect specific allergy to the house dust mites *Dermatophagoides farinae* and *Dermatophagoides pteronyssinus* in atopic dogs

**DOI:** 10.1186/s12917-021-02768-2

**Published:** 2021-02-05

**Authors:** N. Khantavee, C. Chanthick, A. Tungtrongchitr, N. Techakriengkrai, S. Suradhat, N. Sookrung, S. Roytrakul, N. Prapasarakul

**Affiliations:** 1 Department of Veterinary Microbiology, Faculty of Veterinary Science, Chulalongkorn University, 39 Henri-Dunant Road, Pathumwan, Bangkok, 10330 Thailand; 2 Dermatology unit, Veterinary Teaching Hospital, Faculty of Veterinary Medicine, Kasetsart University, 50 Paholyothin Road, Ladyao, Chatuchuk, Bangkok, 10900 Thailand; 3 Department of Parasitology, Faculty of Medicine Siriraj Hospital, Mahidol University, 2 Wanglang Road, Bangkoknoi, Bangkok, 10700 Thailand; 4 Diagnosis and Monitoring of Animal Pathogens Research Unit, Chulalongkorn University, 39 Henri-Dunant Road, Pathumwan, Bangkok, 10330 Thailand; 5 Department of Research and Development, Faculty of Medicine Siriraj Hospital, Mahidol University, 2 Wanglang Road, Bangkoknoi, Bangkok, 10700 Thailand; 6 Proteomics Research Laboratory, National Center for Genetic Engineering and Biotechnology (BIOTEC), Pathum Thani, 12120 Thailand

**Keywords:** Antibody, Biomarker, Canine atopic dermatitis, House dust mites, Immunoglobulin G1 subclass

## Abstract

**Background:**

In dogs with atopic dermatitis, intradermal testing (IDT) or allergen specific IgE serological testing are routinely employed to identify causative allergens. These allergens can then be used for allergen-specific immunotherapy and allergy management. The clinical relevance of this testing is affected by the source of allergen, and other biomarkers that are more related to specific allergens still need to be identified. The aim of this study was to investigate levels of specific IgE, total IgG, and IgG1 and IgG2 subclasses against the local house dust mites (HDM) *Dermatophagoides farinae* (DF) and *D. pteronyssinus* (DP) as biomarkers by using in-house ELISAs in healthy (*n* = 33) and atopic dogs (AD) (*n* = 44) that were either positive or negative by IDT to HDM.

**Results:**

Being over 3 years of age was a risk factor for AD (Odds Ratio (OD) = 4.10, 95% Confidence interval (CI) 1.57–10.75, *p* = 0.0049), but there was no relation to IDT outcomes (OR = 0.9091, 95% CI 0.22–3.74, *p* = 1.00). High levels of all antibody isotypes (IgE, IgG, IgG1 and IgG2) against HDM were found in aged healthy dogs (> 3 years old). In AD, HDM-IgE and IgG1 levels were higher in dogs that were IDT positive to HDM than in IDT negative animals. Levels of IgE and IgG1 could be used to distinguish the specific allergens, whereas total IgG and IgG2 levels were not different between IDT-positive and IDT-negative AD. By the receiver operating characteristic curve at a false-positive rate = 0.10, both IgE and IgG1 showed better sensitivity than IgG and IgG2. Similar to IgE, serum IgG1 concentration was also relevant to IDT outcomes.

**Conclusions:**

Our in-house ELISAs coated with local HDM were useful for evaluating antibody levels, and we propose use of the HDM-specific IgG1 subclass as a biomarker to detect HDM specific allergens in AD, potentially together with an IgE based platform.

**Supplementary Information:**

The online version contains supplementary material available at 10.1186/s12917-021-02768-2.

## Highlights

- Antigens extracted from locally collected house dust mites (HDM) improved the sensitivity and specificity of ELISAs used to detect evidence of exposure to HDM in atopic dogs (AD)
- HDM-specific IgG1 was identified as a useful biomarker to detect HDM allergens in AD, potentially used together with an IgE based platform
- The serological approach to detection of HDM allergy gave less consensual results in dogs over 3 years old.

## Background

Canine atopic dermatitis (CAD) is a major chronic immune-mediated inflammatory and pruritic skin disease, with a genetic predisposition [1]. Underlying allergic reactions can be directed at otherwise harmless substances such as grass, mould spores, house dust mites, and other environmental allergens. Diagnosis of CAD is based on patient history, clinical signs, and elimination of other forms of allergic skin disease. Intradermal testing (IDT) or allergen-specific IgE serological testing (ASIS) are needed to indicate relevant allergens in atopic dogs. This information then can be used to avoid allergen exposure and to formulate allergen-specific immunotherapy (ASIT) [2]. IDT and ASIS still have some problems, as their outcomes can be confounded by factors such as allergen type, allergen source, dog breed, dog age, presence of ectoparasites, and the laboratory techniques used. Different sources of house dust mite extract affect the level of IgE recognition from atopic dog sera [3], and moreover a high background of specific IgE level can be found in some predisposed dog breeds without clinical signs of AD [4]. For practical use by veterinary dermatologists, serological detection is reliable, minimally invasive and convenient to undertake. In serological detection in atopic dogs using allergen-specific IgE and IgGd, compared to IgE, a high IgGd response had a low specificity and was irrelevant to IDT results, implying its uncertain role in atopic dogs [5]. In later studies, the role of allergen-specific IgG in the pathogenesis of CAD was not well defined. IgG is divided into four subclasses (IgG1–4), and certain of these have an interesting relationship to allergic dermatitis [6, 7]. In a previous study, levels of total non-allergen specific IgG1 subclass in sera were affected by parasitic-infestation, atopic dermatitis (AD), and ASIT [7]. In human allergic patients, besides IgE, increases in IgG1 and IgG4 levels were associated with some specific allergens, and also were inducible after ASIT, especially with high IgG4 levels corresponding to relief of symptoms [8]. On the other hand, IgE, IgG1 and IgG4 levels were used as an indicator set to differentiate between non-atopic and atopic dogs, but neither levels of specific immunoglobulins could differentiate both groups of dogs [4]. The model of antigen specific-IgG subclass responses also has been explored in canine leishmaniasis. In relation to different outcomes of leishmania infection, IgG1 levels were noted as a biomarker during the active stage while IgG2 levels were associated with subclinical infection or disease resistance [9]. However, the role of IgG subclasses has not been fully elucidated in CAD.

The aim of the study was to identify an improved biomarker for use in CAD. This involved investigating levels of allergen-specific IgE, total IgG, IgG1 and IgG2 antibody against the local house dust mites (HDM) *Dermatophagoides farinae* (DF) and *D. pteronyssinus* (DP) extracts in healthy dogs and in atopic dogs which had positive or negative IDT reactions to the HDM.

## Results

### Demography of dogs supplying the serum samples

All serum samples were categorized by the source and the dog’s clinical signs. In the AD group, 68% (30/44) of the dogs were positive to HDM (DF and DP) by IDT (Table 1). The number of female and male dogs were similar, and the neuter status was not recorded. Amongst the healthy dogs, those from internal medicine were younger than dogs from the blood bank. In the AD, both sub-groups (+ and – IDT to HDM) were of near median age, and were older than the healthy dogs. Breeds varied in each sub-group, including animals with and without known breed predisposition. In contrast to other sources, Boxer and Rottweiler breeds over three-year-old were the main donors in the blood bank. In AD that were either +IDT or –IDT to HDM, food allergy was found at a rate of about 10–14%. Both sex and age were included in the risk analysis between healthy dogs and AD, but dog breed was not included due to the large number of breeds represented amongst the relatively small number of samples. Sex was not a confounding factor between healthy dogs and AD (OR = 0.58, 95% CI 0.22–1.52, *p* = 0.3359) (Table 2). Being over 3 years of age was a risk factor for AD (OR = 4.10, 95% CI 1.57–10.75, *p* = 0.0049), but there was no relation to IDT outcomes (OR = 0.9091, 95% CI 0.22–3.74, *p* = 1.00) (Table 2).

**Table 1:** Demographic data for the serum samples

Data	Healthy		AD	
Source/ IDT results	Internal medicine	Blood bank	+ IDT to HDM	- IDT to HDM
Number	20	13	30	14
Sex				
-female	11	6	16	5
-male	9	7	14	9
Age range in years (median)	0.17–3.00 (1.00)	2.00–5.00 (3.00)	1.30–15.75 (6.17)	1.16–13.33 (7.08)
Breed	Chihuahua (6), Cross breed (6), Labrador Retriever (3), Siberian Husky (2), Beagle (1), French bulldog (1), Bangkraw (1)	Boxer (7), Rottweiler (5), Golden Retriever (1)	Poodle (7), Shitzu (5), French bulldog (4), Beagle (3), Cross breed (2), Golden Retriever (2), Shiba inu, Labrador retriever, Westy white terrier, Chihuahua, Jack Russel, American Pitbull, Bangkraw (1)	Shitzu (4), Cross breed (4), Pomeranian (3), Bulldog, Golden Retriever, Westy white terrier (1)
Food allergy (%)			10.00	14.28

**Table 2:** Risk factors in healthy dogs and AD from different sources and with different IDT outcomes

Risk factor	Between groups of dogs		
	Healthy (H)	Atopic Dog (AD)	
	Internal medicine and Blood bank	+ IDT and - IDT to HDM	H and AD
Sex			
OR^a^	1.83	2.06	0.58
(95% CI)^b^	(0.47–7.13)	(0.56–7.61)	(0.22–1.52)
*p*^c^	0.4998	0.3419	0.3359
Age > 3 yr			
OR^a^	1025	0.9091	4.103
(95%CI) ^b^	(19.09–55,048)	(0.22–3.74)	(1.57–10.75)
*p*^c^	< 0.0001	1.00	0.0049

^a^Odds ratio

^b^95% Confidence interval

^c^*P*-value; significantly if *p* < 0.05

### Quality of local HDM extracts for coating ELISA plates

**Fig. 1:**
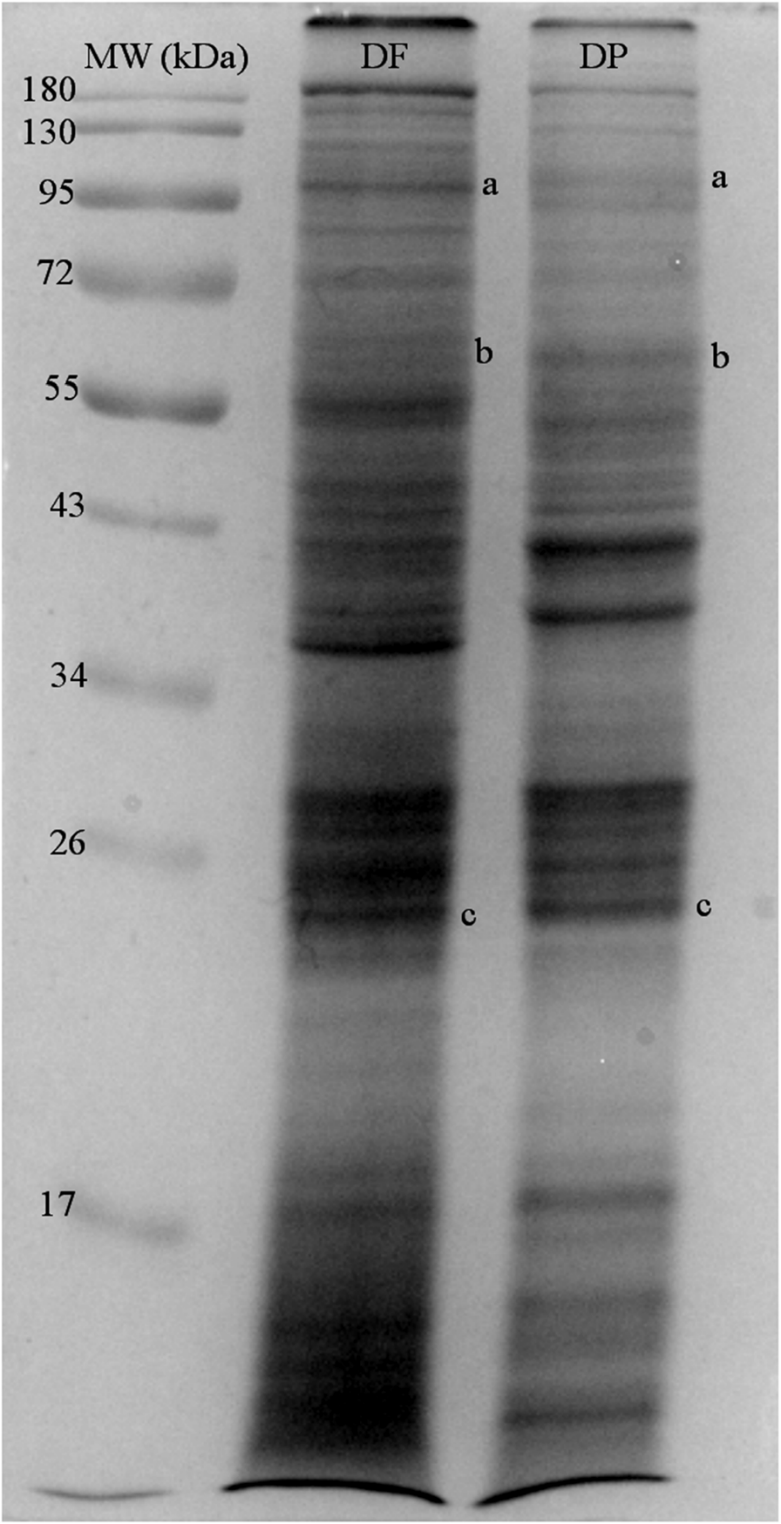
Analysis of protein components in crude extracts of DP and DF (Cropped gel). Protein bands of Der f 15, Der f 18 and Der f/ Der p 1 are located at 97–109 (**a**), 60 (**b**) and 25 (**c**) kDa respectively

HDM extracts contained at least 32 mg of crude protein per gram of purify lived HDM. The concentration of group 1 allergens in DF and DP extracts were 40 and 36.75 μg/ml, respectively. As shown in the cropped electrophoretic gels in Fig. 1, the protein patterns of DF and DP extracts were shown with multiple antigenic bands as previous reports [3, 10]. The results of protein analysis by Tandem mass spectrometry (MS/MS) confirmed that Der f/p 15, 18 and 1 existed in the antigenic bands at 97–109 (a), 60 (b) and 25 (c) kDa, respectively. Data of protein sequences of the HDM allergens are shown in Table 3 with the GenBank accession numbers.

**Table 3:** List of accession number and protein type of each HDM antigenic band

Sources	Size of antigenic bands (kDa)	GenBank No.	Protein types (Biological function)
DF	97–109	AAD52672.1	Der f 15 (Chitinase)
DF	60	AAM19082.1	Der f 18 (Chitinase)
DF	25	AB034946.1	Der f 1 (Preproenzyme)
DP	97–109	AAY84565.1	Der p 15 (Chitinase)
DP	60	AAY84563.1	Der p 18 (Chitinase)
DP	25	AAB60215.1	Der f 1 (Preproenzyme)

### Reproducibility of ELISA

The IgE ELISA for HDM presented an acceptable reproducibility value, and %CV of intra-and inter-assay were 5.4 and 7.1%, respectively. For HDM specific IgG and its subclasses, the % CV of intra-and inter-assays were about 3.0 and 5.8%, respectively.

### DF-specific IgG1 reflects DF allergy in AD

**Fig. 2:**
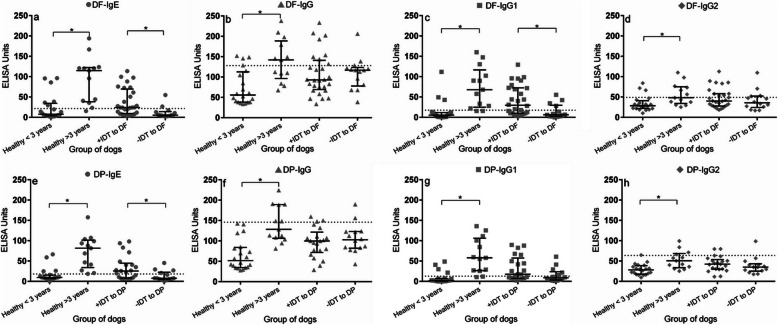
Median and interquartile range of DF- and DP-specific IgE, IgG antibodies and IgG subclasses levels. Aged healthy dogs (> 3 years) had high levels of all antibody types to both species of HDM. In cases of CAD (+IDT or -IDT to DF/DP) high IgE and IgG1 levels were in accordance with IDT results. Dotted line; cut-off levels for each ELISA

Aged healthy dogs had significantly higher DF specific-IgE levels than both younger healthy dogs and AD (Fig. 2a). Considering the groups by IDT outcomes, high levels of DF specific-IgE measured by ELISA were consistent with DF allergy detected by IDT. Nevertheless, elevated DF-specific IgE levels were also found in healthy dogs. The aged healthy dogs had much higher levels of DF-specific total IgG than they did to the other antibodies (Fig. 1b), which were not different between healthy and AD or HDM allergen types.

For DF-specific IgG1 levels, the aged healthy dogs showed the highest median levels compared to the other groups (Fig. 2c). In the AD group, DF-IgG1 levels were significantly higher in AD that were positive to DF by IDT (+IDT to DF) compared to those that were negative (−IDT to DF). Interestingly, the patterns of DF-IgE (Fig. 2a) and DF-IgG1 (Fig. 2c) closely corresponded. In contrast, levels of the DF-specific IgG2 subclass were not different between AD whether they were positive or negative to DF, and healthy dogs still showed a high median level (Fig. 1d).

Similarly, the pattern of antibody levels specific to the DP antigen (Fig. 2e-h) reassembled the response to DF as well as to the pattern of DF specific detection (Fig. 2a-d). The aged healthy dogs showed the highest levels for all detected markers. According to the cut-off values, IgE and IgG1specific to DP could distinguish between healthy dogs and AD that had positive results to DP by IDT, and between AD (+IDT to DP) and AD (−IDT to DP) (Fig. 2e and g).

### Ratio of DF- or DP-IgG1/IgG enhances the validity of allergic detection in AD

**Fig. 3:**
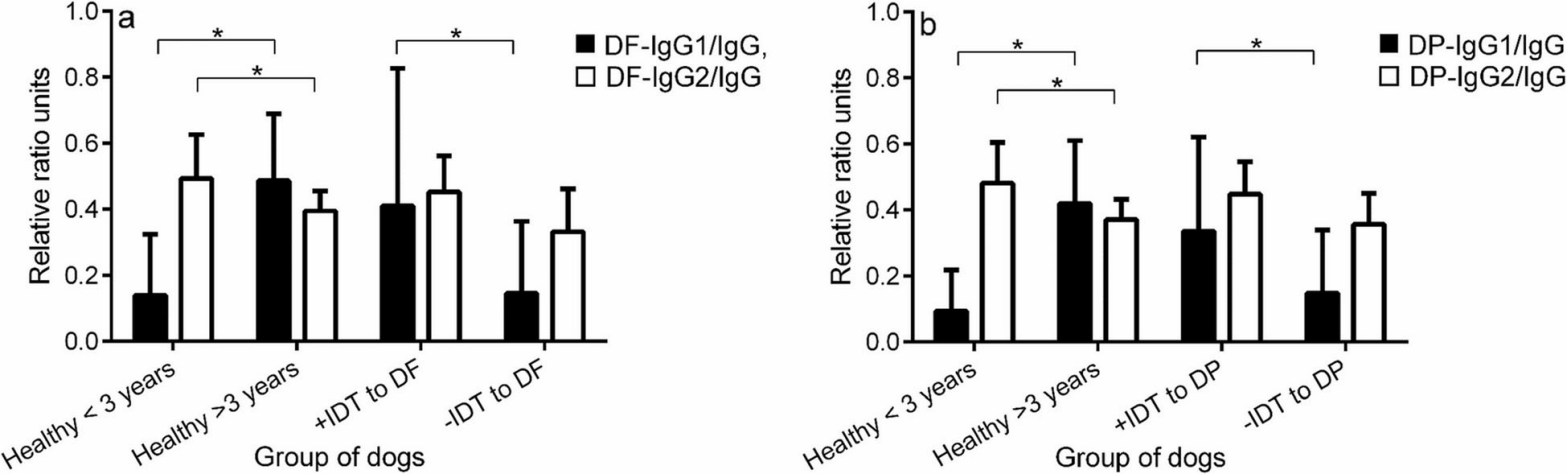
The pattern of specific IgG1 and IgG2 in total IgG against DF and DP in healthy and AD dogs. A low IgG1/total IgG ratio and high value for the IgG2/total IgG ratio were observed in the young healthy dogs (< 3 years) and in AD with negative IDT to DF/DP, whereas the patterns were switched in the aged healthy dogs (> 3 years). AD that were positive in IDT to DF/DP showed a high ratio of both IgG1/total IgG and IgG2/total IgG

The ratios of DF-IgG1/IgG and IgG2/IgG were calculated to reduce the individual confounder of their total specific IgG difference. A low DF-IgG1/IgG ratio and high value for the DF-IgG2/IgG ratio were observed in the young healthy dogs whereas the patterns were switched in the aged healthy dogs (Fig. 3a). The ratio of DF-IgG1/IgG was significantly increased in AD that were DF positive by IDT (AD+IDT to DF), but this was not seen for DF-IgG2/IgG.

The DP-IgG1/IgG ratio level was higher in the aged healthy group than in the young group (Fig. 3b), as with the results from the DF panel. These ratios also showed a significantly higher level in AD (+IDT to DP) than in AD (−IDT to DP), while in certain samples detection just with DP-IgG1 levels could not separate between AD (+IDT and -IDT to DP) (Fig. 2g).

### HDM-specific IgG1 has similar sensitivity as HDM-specific IgE

**Fig. 4:**
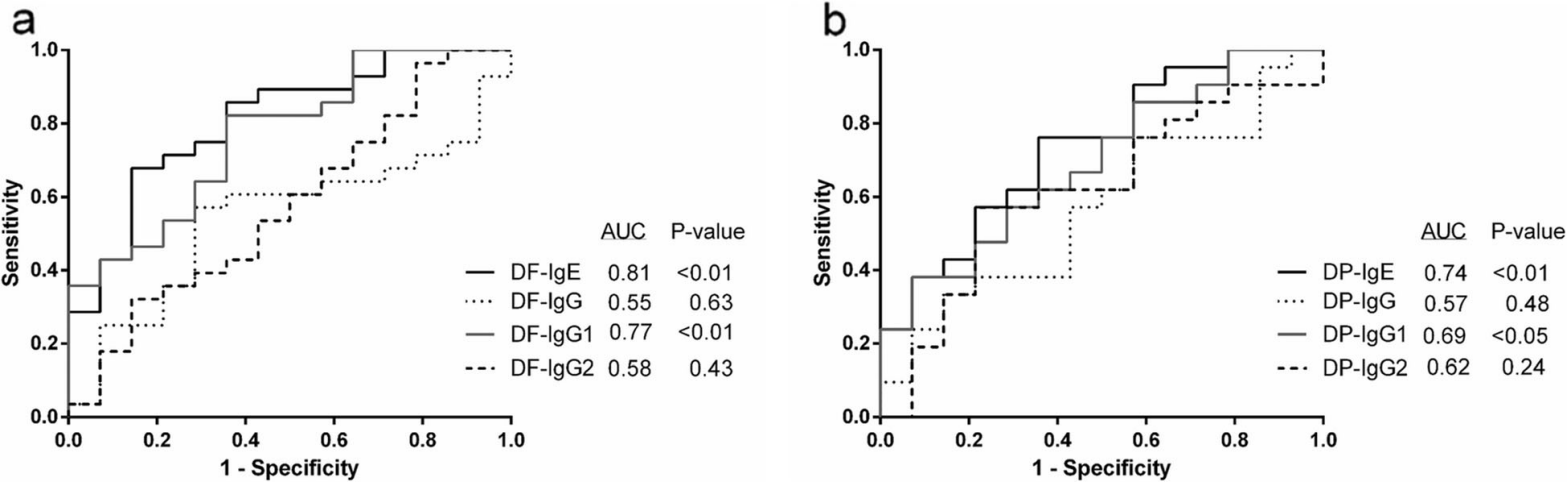
Receiver operating characteristic curve (ROC) for prediction of DF and DP allergy based on the antibody levels measured by enzyme-linked immunosorbent assay. By IgE and IgG1 subclass, the curve analysis shows prediction accuracy with an area under the curve (AUC) (*P* <  0.05)

By using our in-house ELISAs, the sensitivity and specificity values achieved for the different antibody classes and antigens varied considerably as shown by the receiver operating characteristic curve (ROC) (Fig. 4). For confirming DF allergy, at a false-positive rate = 0.10, either IgE or IgG1 showed better sensitivity than IgG and IgG2 and also showed the significant AUC (Fig. 4a). For DP allergy, IgE and IgG1 showed the significant AUC, and gave better sensitivity than IgG and IgG2 at the same point of false-positive rate (Fig. 4b). Regarding the limitation of low samples of AD dog, the mean + 4SD of negative control was used as the cut-off level and used to re-calculate the sensitivity and specificity of HDM allergy, the result was shown in Supplementary Table 1. By assessment agreement between IDT and ELISA, both IgE and IgG1 against both HDM antigens showed a similar value of agreement (Table 4), whereas the agreements for IgG or IgG2 against IDT were poor (k = 0.057 and 0.078, respectively).

**Table 4:** Agreement analysis between IDT and in-house ELISA with different type of specific immunoglobulins

Allergen	Agreement between IDT and ELISA (k^a^/strength of agreement^b^)			
	IgE	IgG	IgG1	IgG2
DF	0.400/fair	0.108/poor	0.286/fair	0.057/poor
DP	0.318/fair	0.086/poor	0.253/fair	0.078/poor

^a^kappa value

^b^strength of agreement, the interpretation if kappa value =0.00–0.20 (poor); 0.21–0.40 (fair); = 0.41–0.60 (moderate); = 0.61–0.80 (substantial); = 0.81–0.99 (almost perfect)

## Discussion

In this study we explored levels of specific IgE, IgG, IgG1 and IgG2 antibody against local sources of DF and DP extracts in healthy dogs and in AD by using in-house ELISAs. IgE levels measured by ELISA were not satisfactory as a screening test without clinical diagnosis, because healthy dogs still presented high levels of DF- and DP- IgE (Fig. 2a and e). In general, age, sex and breed have an impact on allergen-specific IgE production [11]. In our study, healthy dogs over 3 years old had a greater likelihood of presenting high levels of DF- and DP-IgE than did younger dogs, in agreement with finding in previous studies [4, 11]. These high levels might be associated with exposure to natural ectoparasites, and thus the age of the patient should be considered as part of the interpretation of results [12]. In this study, the breed was not included in the risk analysis because of the small numbers of animals available in each breed type. However, Boxers and Rottweilers over 3-year-old were the major population, and these showed quite high levels of intrinsic background IgG and IgE. A previous study reporting that Boxers and Rottweilers have higher serum IgE level against HDM than other breeds [11]. This observation confirmed that false positive results could be found in aged-dogs or in some predisposed breeds. Moreover, some AD dogs that were positive to HDM had IgE levels lower than the cut-off. However, the specific IgE related to HDM type was detectable in dogs with atopic dermatitis (Fig. 2 a and e) and this response was in accordance with their IDT outcomes.

In this study, both HDM extracts were prepared from the Siriraj Dust Mite Center for Services and Research (SDMC). The HDM extracts especially standard groups 1 allergen extracts (Der f 1 and Der p 1) [13] are routinely used for diagnosis in patients with allergic rhinitis and in allergic field research. The concentration of group 1 allergens in DF and DP extracts was adequate as specified for FDA reference preparations [14], and the important allergens of HDM like Der f/p 15 (98–105 kDa) and Der f/p 18 (60 kDa) were confirmed their existence in our extracts by Liquid Chromatography-Mass Spectrometer (LC-MS/MS) [10, 15, 16]. This finding supported that our HDM source have the quality for allergy testing in atopic dogs. Regarding the crude protein extracts, the cross-reactivity between HDMs and other invertebrates, and the variety of allergenic components could affect immunoglobulin E levels [3, 17, 18]. The proper protein components of DF and DP extracts related to allergic conditions in dogs should be further investigated to improve sensitivity and specificity.

In addition to the IgE level, the finding of high levels of HDM–specific IgG, IgG1 and IgG2 among the aged healthy group confirmed the need for caution about background antibody levels: although the antibodies were specific to HDM the latter still consist of a number of proteins [19]. Conversely, the levels of DF- or DP-IgG1 could be used to identify the type of HDM allergen in AD. The sensitivity results for the HDM-IgG1 ELISA were the same as the sensitivity with the IgE ELISA at a false-positive rate = 0.10 (Fig. 4). Moreover, the IgG1 ELISA showed the same strength of agreement to IDT as did IgE (Table 4). These results suggested that the specific IgG1 concentration could identify HDM allergens whilst using about 100 times less serum than needed for IgE. Use of low volumes of blood is beneficial in term of animal welfare, and the test can be duplicated. Moreover, specific IgG1 seemed to play a role in immunopathogenesis of atopic dermatitis.

Regarding the IgG subclass volume in dogs, the proportions of IgG1 and IgG2 are approximately equal and deviate during episode of inflammation; for instance, high IgG2 levels occur in furunculosis, otitis externa and in autoimmune haemolytic anaemia [6]. Varying background amounts of immunoglobulins in each individual dog could be a major drawback leading to mis-interpretation in serological detection. To circumvent this problem, measuring the ratio of IgG1/ total IgG and IgG2/ total IgG should reduce any individual bias. The results of this study confirmed the validity of this approach, with the proportions of both *D. farinae*- and *D. pteronyssinus*-specific IgG1/total IgG reflecting true positives. In contrast, the IgG2/ total IgG levels clearly distinguished the healthy and HDM-IDT negative group from the HDM-IDT positive group, indicating an increase in specificity. A bias towards IgG1 is believed to occur as a response in TH2-mediated disease in mice and dogs, especially in the case of canine leishmaniasis [9, 20]. On the other hand, a predominance of Th1 cytokines and *Leishmania chagasi*-specific IgG2 was present in vaccinated dogs or asymptomatic dogs exposed to this protozoan [21]. Our findings imply that an IgG1 response may reflect a type 1 hypersensitivity triggered by specific HDM allergens, whereas IgG2 levels may represent a predominance of Th1 cytokines. Moreover, the bias towards allergen specific IgG1/IgG2 could enhance diagnosis for AD, and could help to predict the outcome of ASIT.

In this study, IDT was used as the gold standard, but associated technical problems were not fully evaluated in clinical samples. Regarding the agreement between IDT and ELISA, serological detection might serve as a tool for specific allergen identification as confirmed by using HDM allergenic model, but this was not recommended for investigation in healthy dogs. In Thailand, IDT is widely used because the commercial ELISA is costly and time-consuming. Identification of DF allergy through our IgE ELISA had a similar sensitivity to a commercial IgE ELISA using high affinity Fc-epsilon receptor alpha chain protein (FcεRIα) [22], this commercial test is very specific to canine IgE. The IgE assay in this study used the commercial monoclonal anti-dog IgE antibody to detect the level of allergen-specific IgE. The specificity of antibody to dog IgE without cross reaction to dog IgM, IgA, IgG1 and IgG2 was claimed by manufacture and it has been used in many previous reports [23–25].

Interestingly, the DP-IgE ELISA in this study showed better sensitivity than that commercial test. Different sources of DP might influence the results of sensitivity [3], and locally prepared DP extracts are more likely to be recognized by AD dogs in the same area. The agreement between results of IDT and IgE ELISA in this study was only fair, even though the same source of HDM was used. Previously, the correlation/agreement between FcεRIα-based ELISA assay and IDT also showed in poor and moderate level, respectively [22, 26]. This phenomenon might be affected by different forms of IgE: IgE on skin has a longer life-span than that in the circulation [27], so this may explain why some AD (+IDT) had lower levels of HDM-specific IgE and a fair agreement was found between IDT and IgE ELISA results. There is still little evidence in support of an immunopathological role in respect of the immunoglobulin isotype. Besides, the specific IgE detection and source of allergen, geographical difference or background of allergic exposure in dog patients should be taken into consideration of the correlation/agreement [22, 28, 29].

There has been a lack of information on the role of IgG1, which may act as another allergic marker, or possibly function as a blocking antibody after an IgE response. In humans with allergic rhinitis, IgG1 production depends on the frequency of protein exposure, and its response becomes dominant after ASIT [8]. The lack of difference in DF-IgG1 levels found between AD and healthy dogs in this study was consistent with the results of a previous study [4], but a relationship between aging and IgE and IgG1 titers was found. Moreover, if the positive or negative results from IDT against DF or DP were considered in the inclusion criterion this could well enhance the specificity of the results. Nevertheless, we suggest that levels of IgG1 and IgG2 could be used for longitudinal monitoring of responses in different stages of AD or for HDM specific immunotherapy.

## Conclusions

Levels of IgG1 and IgE had similar patterns during episodes of HDM allergy in CAD. An in-house ELISA using IgG1 could be used to differentiate between DF or DP allergy, and showed the similar trend as IgE detection. Unfortunately, this serological approach gave less consensual results in dogs over 3 years old. The detection platform for HDM allergy identification using IgG1 and ratio of IgG1/IgG was affordable, valid, and only required a very small serum sample.

## Methods

### Serum samples

A total of 33 healthy dogs were enrolled from the Chulalongkorn University blood bank, with the following inclusion criteria: normal at physical examination; not suffering from any diseases; no previous history of skin problems; and normal skin appearance. Serum samples were collected and stored at − 20 °C until used.

Our AD subjects were enrolled from the Veterinary Teaching Hospital, Faculty of Veterinary Medicine, Kasetsart University. CAD was defined in 44 dogs by a combination of their clinical histories, clinical signs, match to Favrot’s criteria [30], ruling out other pruritic skin disease, and little improvement of skin condition after 8 weeks of dietary restriction using a hypoallergenic food or a novel protein [2]. The AD subjects were not previously prescribed with any steroid regimen. IDT was undertaken in all AD cases after a withdrawal period of at least 2 weeks for anti-inflammatory and anti-pruritic drugs. IDT was performed according to the standard protocol recommended by the manufacturer, using 45 allergen extracts from ALK Abello (ALK Abello), excluding DF and DP. Commercial HDM extracts for IDT were obtained from Siriraj House Dust Mite Center (SDMC) (Mahidol University, Thailand). Allergen extracts were diluted as recommendation by the manufacturer. After interpretation with the same criteria as in a previous report [31], AD cases were divided into IDT negative and IDT positive to HDM (for DF and DP). Serum samples were collected on the same day as IDT testing and were stored at − 20 °C until used.

### Preparation of HDM extract

Purified DF (batch no. DF-SDMC 080158) and DP (batch no. DPT-BKK 060158), both with > 99% purity, were obtained from SDMC. Two grams of each mite had been individually resuspended in 8 mL of 0.01 M phosphate buffered saline (PBS), pH 7.4 and then were homogenized using an Omni Sonic Ruptor 4000 (Omni International) at 35% amplitude with 0.5 cycles (15 min, on ice). The supernatant was collected after centrifugation at 12,000 x *g*, 4 °C for 5 min (Sorvall Legend X1R, Thermo Scientific). Composition analysis of extracts were performed for quality control. All the processes of validation and production of DF and DP extracts were executed following good manufacturing practice (GMP) regulations, under the supervision of an SDMC specialist. Protein content was measured by Bradford’s assay (Protein Assay Kit II, Bio-Rad), and the supernatant was kept in aliquots at − 20 °C until used. Antigenic bands of HDM extracts were checked by SDS-PAGE, as previous reported [10]. To confirm the existence of major allergens, Der f/p 1, 15 and 18, in the local HDM sources, the antigenic bands at 97–109 (a), 60 (b) and 25 (c) kDa of DF and DP extracts were analyzed by LC-MS/MS. [10] In additional, HDM extracts were examined for adequate content of the major allergens Der f 1 and Der p 1 using commercial ELISAs (Der f 1 ELISA kit (6A8/4C1) and Der p 1 ELISA kit (5H8/4C1), Indoor biotechnologies).

### In-house DF/DP-specific IgE ELISAs

Pooled serum samples from five AD dogs and five young healthy dogs were used in a checker-board titration. AD dogs were selected for their strongly positive IDT (+ 4) to DF/DP from commercial (ALK Abello and Greer Labs Inc) and SDMC allergens and also their positive results to both DF and DP in a commercial ASIS (Avacta Animal Health). Sera from young healthy dogs (6-8 months old) negative by commercial ASIS were used as negative controls. DF and DP extracts were separately prepared in 0.2 M sodium carbonate-bicarbonate buffer, pH 9.4 (BupH™ Carbonate-Bicarbonate Buffer Packs, Thermo Fisher Scientific). One hundred microliters of HDM solution was added into the wells of 96-well plates (Costar 3590 EIA, Corning) and incubated overnight at 37 °C. After washing with washing buffer consisting of PBS containing 0.05% Tween 20 (Affymetrix, Fisher Scientific) (PBST), 200 μl of blocking buffer (PBST containing 1% Bovine serum albumin (BSA)) was added and incubated at 37 °C for 1 h. Plates were rinsed before adding 100 μl of diluted pooled serum samples in duplicate, consisting of two-fold serial dilutions in blocking buffer starting at 1/5. Plates were incubated at 37 °C for 2 h before washing. Monoclonal anti-dog IgE (clone E6-2A1, Serotec/Bio-Rad) diluted in blocking buffer (1/2000) were added and incubated at 37 °C for 1 h. After washing, 100 μl of a phosphatase conjugate (1/2000 in blocking buffer) (goat anti-mouse IgG & Human ads-alkaline phosphatase, Southern biotech) was added, incubated at 37 °C for 1 h, and washed as before. Alkaline phosphatase substrate (Sensitest Canine IgE Substrate, Avacta Animal Health Limited) was added at 25 °C for 20 min, and plates were immediately read at an optical density (OD) of 450 nm using an ELx808 ultra microplate reader and KC4 3.3 Rev. Ten software (Bio-Tek instruments Inc.). Results were subtracted with the OD of the blank control before generating titration curves. The middle point of the near-linear part was selected as an optimal point. The optimal HDM concentration for coating was 20 μg/mL with a serum dilution of 1/5. The reaction was quantified in ELISA units to act as a reference, as s previously described [32]. Results from a pooled serum sample from five AD dogs was used for calibration, and arbitrarily set at 100 units (U). A cut-off value was established by using the mean + 4 standard deviations of the negative control samples. For intra- and inter assay consistency, results of detection were acceptable with a coefficient of variation (CV) not exceeding 10%.

### In-house DF/DP specific-IgG, IgG1 and IgG2 ELISAs

Checker-board titrations for the other antibody classes were performed as described for the IgE ELISA, with some modifications. Three different peroxidase conjugates were used to detect allergen-specific IgG, IgG1 and IgG2, included polyclonal anti-dog IgG (IgG antibody (AAI32P), Bio-Rad.), polyclonal anti-dog IgG1 (IgG1 antibody (AHP947P), Bio-Rad), and polyclonal anti-dog IgG2 (IgG2 antibody (AHP948P), Bio-Rad), respectively. These conjugates were individually diluted in blocking buffer at 1/4000 before use. Peroxidase substrate (ABTS® peroxidase substrate, KPL Inc.) was added for colour development. The optimal serum dilution was 1/500.

### Data and statistical analyses

GraphPad Prism (GraphPad Software Incorporated) was used for statistical analyses. Risk factors were analyzed in the sample population. Levels of DF- and DP- specific immunoglobulin between healthy and AD dogs were determined for normality by the D’Agostino & Pearson omnibus, and medians were compared by the Kruskal-Wallis. The Dunn’s multiple comparisons test was applied for post doc analysis. Differences were considered significant if *p* values were less than 0.05. The validity of each antibody isotypes to indicate HDM allergy in AD dogs was analyzed by ROC curve, and AUC considered their significant if *p* values were less than 0.05.

## Supplementary Information

**Additional file 1: Supplementary Table 1.** Sensitivity and specificity (%) of in-house ELISAs through different type of specific immunoglobulins.

## Data Availability

The datasets of protein sequences generated and/or analyzed during this study are available in the GenBank and the accession numbers are AAD52672.1, AAM19082.1, AB034946.1, AAY84565.1, AAY84563.1 and AAB60215.1.

## Abbreviations

AD: Atopic dogs
CAD: Canine atopic dermatitis
DF: *Dermatophagoides farinae*
DP: *Dermatophagoides pteronyssinus*
HDM: house dust mite
IDT: Intradermal skin test
