# Supplementary material for: Immunoglobulin G1 subclass responses can be used to detect specific allergy to the house dust mites Dermatophagoides farinae and Dermatophagoides pteronyssinus in atopic dogs
Source: BMC Vet Res. 2021 Feb 5;17:71. doi: 10.1186/s12917-021-02768-2 (PMC7866685; doi:10.1186/s12917-021-02768-2)
Supplement: Supplementary file 1 — Additional file 1: Supplementary Table 1. Sensitivity and specificity (%) of in-house ELISAs through different type of specific immunoglobulins. [file 12917_2021_2768_MOESM1_ESM.docx]

**Supplementary table 1**

Sensitivity and specificity (%) of in-house ELISAs through different type of specific immunoglobulins

| Allergen | Number  IDT+ | Number IDT - | Sensitivity (%) | | | | Specificity (%) | | | |
| --- | --- | --- | --- | --- | --- | --- | --- | --- | --- | --- |
|  |  |  | IgE | IgG | IgG1 | IgG2 | IgE | IgG | IgG1 | IgG2 |
| DF | 28 | 14 | 60.7 | 28.6 | 60.7 | 35.7 | 85.7 | 85.7 | 71.4 | 71.4 |
| DP | 21 | 14 | 61.9 | 14.3 | 61.9 | 9.52 | 71.4 | 92.9 | 64.3 | 92.9 |
